# Distinct tissue-specific transcriptional regulation revealed by gene regulatory networks in maize

**DOI:** 10.1186/s12870-018-1329-y

**Published:** 2018-06-07

**Authors:** Ji Huang, Juefei Zheng, Hui Yuan, Karen McGinnis

**Affiliations:** 1 Department of Biological Science, Florida State University, Tallahassee, Florida 32306 USA; 2 School of Life Sciences, Tsinghua University, Beijing, 100084 China; 3 Department of Statistics, Florida State University, Tallahassee, Florida 32306 USA

**Keywords:** Maize, Gene expression, Transcriptional regulation, Transcription factor, Network, Bioinformatics, Systems biology, Machine learning, Database

## Abstract

**Background:**

Transcription factors (TFs) are proteins that can bind to DNA sequences and regulate gene expression. Many TFs are master regulators in cells that contribute to tissue-specific and cell-type-specific gene expression patterns in eukaryotes. Maize has been a model organism for over one hundred years, but little is known about its tissue-specific gene regulation through TFs. In this study, we used a network approach to elucidate gene regulatory networks (GRNs) in four tissues (leaf, root, SAM and seed) in maize. We utilized GENIE3, a machine-learning algorithm combined with large quantity of RNA-Seq expression data to construct four tissue-specific GRNs. Unlike some other techniques, this approach is not limited by high-quality Position Weighed Matrix (PWM), and can therefore predict GRNs for over 2000 TFs in maize.

**Results:**

Although many TFs were expressed across multiple tissues, a multi-tiered analysis predicted tissue-specific regulatory functions for many transcription factors. Some well-studied TFs emerged within the four tissue-specific GRNs, and the GRN predictions matched expectations based upon published results for many of these examples. Our GRNs were also validated by ChIP-Seq datasets (KN1, FEA4 and O2). Key TFs were identified for each tissue and matched expectations for key regulators in each tissue, including GO enrichment and identity with known regulatory factors for that tissue. We also found functional modules in each network by clustering analysis with the MCL algorithm.

**Conclusions:**

By combining publicly available genome-wide expression data and network analysis, we can uncover GRNs at tissue-level resolution in maize. Since ChIP-Seq and PWMs are still limited in several model organisms, our study provides a uniform platform that can be adapted to any species with genome-wide expression data to construct GRNs. We also present a publicly available database, maize tissue-specific GRN (mGRN, https://www.bio.fsu.edu/mcginnislab/mgrn/), for easy querying. All source code and data are available at Github (https://github.com/timedreamer/maize_tissue-specific_GRN).

**Electronic supplementary material:**

The online version of this article (10.1186/s12870-018-1329-y) contains supplementary material, which is available to authorized users.

## Background

Regulation of gene expression is one of the most important and complex issues in biology. It is particularly interesting and intricate in eukaryotic species due to their large genomes and higher-order nuclear organization. Plant biologists pioneered genetic research in gene regulation, from Gregor Mendel to Barbara McClintock, and their work forms the foundation of the current understanding.

Maize (*Zea mays*) has been a model organism for over a hundred years, and is also of substantial economic significance. The recent development of next-generation sequencing has greatly enhanced maize research by making it easier to investigate genome-wide expression changes. Such data can be used to construct gene regulatory networks (GRNs) that elucidate gene regulation interactions in a systematic way [1, 2]. Even though all cells carry the same genetic code, cellular differentiation is likely guided by distinct GRNs. There has been limited research in maize to decipher tissue-specific GRNs [3, 4].

Although there are many different types of genetic regulatory proteins, transcription factors are of particular interest because they represent a relatively straightforward regulatory interaction between a protein and the chromosome, likely leading to direct changes in transcriptional activity. One of the TF resources in maize is the Grass Regulatory Information Services (GRASSIUS) with 2587 annotated TFs in maize [5], and 2034 TF open reading frame (ORF) cloned vectors [6] to facilitate TF-target interaction analysis. In this study, we focused on the TFs from the GRASSIUS annotation and our GRN refers to the interactions between the GRASSIUS TFs and their regulated targets. Other types of regulation, such as protein-protein interaction and epigenetic regulation, are beyond the scope of this study but can be analyzed with variations on the approaches available through GRN analysis [7, 8].

To unravel TF regulatory interactions, in vivo methods using chromatin immunoprecipitation (ChIP) are the gold standard. Basically, ChIP experiments isolate TF-DNA complexes in vivo. Coupled with PCR (ChIP-qPCR), microarray (ChIP-chip) or sequencing (ChIP-Seq), this approach can determine the positions of TF binding and allow the prediction of high-confidence TF regulatory regions. However, in plants there are a relatively small number of published ChIP datasets, perhaps due to limitations with plant transformation, antibody efficiency, and other experimental difficulties. Even in a well-studied plant model like *Arabidopsis thaliana*, there are only 46 TFs with ChIP-chip/Seq data, collected from three main databases including JASPAR [9], CIS-BP [10] and CressInt [11]. In maize, there is published ChIP-Seq data for only five TFs. As a comparison, the human ENCODE project generated ChIP-Seq data for 630 TFs [12].

As an alternative or complementary approach, in vitro methods could be applied to construct large-scale GRNs. Several yeast-one hybrid (Y1H) systems have been established for Arabidopsis TF-DNA screening [13–15]. In maize, the TFome project [6] provides an invaluable resource of over 2000 maize TF clones to facilitate high-throughput studies, including a recent Y1H screen that identified over a thousand TF-Target interactions in a maize phenolic metabolic pathway [16]. Other potential data for GRNs can be generated with systematic evolution of ligands by exponential enrichment (SELEX) [17], protein binding microarrays (PBM) [18] or DNA affinity purification sequencing (DAP-seq) [19]. These methods can discover *cis*-elements for tens to hundreds of TFs that help decode complex transcriptional networks. DAP-Seq can also incorporate DNA methylation information which has been shown to impact TF-target binding in Arabidopsis [19] and human [20]. Most of these approaches have not yet been used in maize.

PlantRegMap and some other tools [21–23] predict TF binding sites based upon the idea that homologous TFs in different related species might recognize the same motif or *cis*-regulatory element (CRE). These sequences are represented in Position Weighted Matrices (PWMs) that can be used to predict TF targets. This approach relies on high-quality PWMs generated from ChIP-Seq, PBM or DAP-Seq data. PlantRegMap collected 674 high-quality motifs which could project to only 229 of the 2587 TFs predicted for maize by GRASSIUS [5]. Another limitation of in vitro and homology-based methods is that regulatory interactions at tissue and cell-type levels cannot be detected or inferred from this data alone.

An alternative approach to infer regulatory networks is through the use of statistical inference algorithms applied to gene expression data. One particularly effective algorithm is GENIE3 [24], which was the highest scoring of inference algorithms that were compared based upon the Dialogue for Reverse Engineering Assessments and Methods (DREAM) challenge 4 and 5 [25]. GENIE3 has been successfully applied to Arabidopsis [26] and maize [27] GRN construction. This method uses regression trees [28] to model regulators for each gene, and can therefore predict TF-target interactions. GENIE3 it does not require a specific experimental design, and can therefore be applied to the large amount of publicly available genome-wide expression dataset. Furthermore, GENIE3 can reveal non-linear relationships. It makes use of the random forest algorithm [29] with ensembling 1000 bootstrap trees and finds the regulators that can reduce model variance by splitting trees. GENIE3 has implementations in Python, Matlab and R languages that are easy for researchers to use. Also, similar methods have been adapted to time-series [30], single-cell [31], and integrated [32] GRNs showing its wide applicability. GENIE3 takes advantage of parallel computing and can generates large networks on a multi-core desktop. By using network analysis, GRNs were constructed for a total of 2241 TFs in four different tissues (leaf, root, Shoot Apical Meristem and seed). We discovered TF interactions that could be confirmed by ChIP-Seq datasets, which suggest that this approach was effective at predicting true interactions. GRNs in different tissues revealed tissue-specific TF regulatory interactions that could correlate with distinct biological functions. We found the centrality of a TF didn’t correlate with its expression and each tissue employed distinct TFs as master regulators. A user-friendly web portal (http://www.bio.fsu.edu/mcginnislab/mgrn) was developed. All source codes are available at Github and can be easily applied to other organisms.

## Results

### Maize transcription factors show tissue-specific expression patterns

**Fig. 1:**
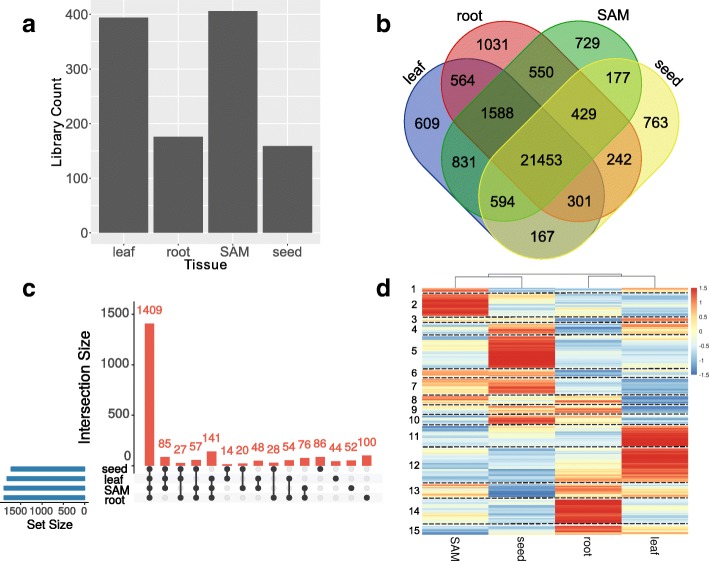
Maize Gene Regulatory Networks (GRNs) in four tissues. **a** Number of RNA-Seq libraries used to build GRNs in each tissue. Libraries were grouped into tissues based on SRA metadata data and/or published papers (details in Supplemental table1). SAM: shoot apical meristem. **b** A Venn diagram showing the overlap of genes expressed in leaf, root, SAM and seed. A gene was designated as expressed in a tissue if it had counts per million (CPM) value higher than 1 in more than 10% of libraries. **c** An UpSet graph showing the overlap of expressed Transcription Factors (TFs) in each tissue. Number of TFs expressed in each individual or combination of tissues were represented in bar plot (orange). The intersections were represented by connected black dots. Total number of TFs expressed in each tissue were represented in blue bar plot. **d** Expression heatmap of 1409 TFs that were expressed in all four tissues. TFs were clustered into 15 sub-groups (separated by dashed lines) based on their expression patterns. Gene expression value from each tissue were averaged and z-transformed, resulting in a scaled expression values between − 1.5 and + 1.5 for each gene. Hierarchical Clustering was calculated by hclust() function in R

Previously, we re-analyzed 1266 high-quality RNA-Seq libraries in various maize tissues and generated a gene coexpression network [33]. Tissues with more than 100 libraries from that expression matrix were chosen to construct tissue-specific GRNs (Fig. 1a). There were four tissues included: leaf, root, shoot apical meristem (SAM) and seed (Additional file 1). In each tissue, genes having more than one counts per million (CPM) in more than 10% of tissue libraries were considered expressed in that tissue. We found 76.06% (30,028/39479) of genes in maize were expressed in at least one tissue, and 54.34% (21,453/39479) of genes were expressed in all four tissues (Fig. 1b). These numbers are comparable with a previous study [34] that reported 91.4% of genes were expressed in at least one tissue and 44.5% were expressed in all tissues, although our analysis used fewer tissues and an updated gene annotation (AGPv3). A small portion of genes exhibited tissue-specific expression (Fig. 1b), and gene ontology (GO) enrichment analyses were conducted for those genes (Additional file 2). We found leaf-specific genes were enriched for photosynthesis activity (*p*-value = 2.40E-06) and seed-specific genes were enriched in nutrient reservoir activity (*p*-value = 1.39E-37), including 20 zein genes.

Next, we inspected the expression pattern of TFs among four tissues. Of all 2587 TFs annotated by GRASSIUS [5] in maize AGPv3, 86.63% were expressed in at least one tissue and 54.46% (1409/2587) were expressed in all four tissues in our data (Fig. 1c). This suggests that a considerable number of TFs are present in multiple plant organs. 10.90% (282/2587) of TFs were only expressed in one tissue (44 in leaf, 100 in root, 52 in SAM and 86 in seed), including some well-studied examples: *Narrow Sheath1* (NS1) in leaf; *Rootless concerning crown and seminal roots1* (RTCS1) in root; *Teosinte Branched1* (TB1) in SAM; *Viviparous1* (VP1) in seed (Table 1). Mutants of these TFs have been shown to exhibit phenotypes in relevant tissues. For example, the *ns* mutant plants exhibit deletion of lower leaf margins [35]; the *rtcs1* mutants completely lose crown and lateral roots [36]; *tb1* mutants are highly branched due to the loss of apical dominance [37]; *vp1* mutant seeds germinate early on immature cobs [38]. Even though some TFs were expressed in all four tissues, these 1409 genes had distinct expression patterns (Fig. 1d), which may contribute to tissue-specific functions. Since TFs are pivotal gene expression regulators, their patterns may represent diverse and tissue-specific gene regulatory networks.

**Table 1:** Examples of TFs that are unique to a single tissue

Tissue	Gene name	GeneID	Reference
Leaf	Narrow sheath1	GRMZM2G069028	[35]
Root	Rootless concerning crown and seminal roots1	GRMZM2G092542	[82]
SAM	Teosinte branched1	AC233950.1_FG002	[83]
Seed	Viviparous1	GRMZM2G133398	[84]
Seed	Opaque endosperm2	GRMZM2G015534	[43]
Seed	Prolamin-box binding factor1	GRMZM2G146283	[85]
Seed	Transfer cell response regulator1	GRMZM2G016145	[86]

### Gene regulatory network construction for four tissues

To construct tissue-specific GRNs, we used GEne Network Inference with Ensemble of trees (GENIE3) algorithm [24], the best performer in the DREAM 4 and 5 challenge that used a tree-based ensemble machine learning method to predict gene regulatory relationships [25]. Although extensive benchmark comparisons between this and other algorithms have been reported previously [24, 25], we compared GENIE3 with two other *state-of-art* algorithms: Minimum Redundancy NETwork (MRNET) [39] and context likelihood of relatedness (CLR) algorithm [40]. The tissue-specific GRNs were inferred from each tissue’s expression matrix by setting the 2587 TFs as “candidate regulators”. This resulted in a predicted GRN for each of the 4 tissue types.

First, we evaluated the quality of these four networks by using published TF ChIP-Seq data of DNA precipitated using antibodies that would interact with Knotted1 (KN1) [41], Fasciated ear4 (FEA4) [42] and Opaque endosperm2 (O2) [43]. These 3 proteins are known TFs with specialized function in SAM and ear development (KN1 and FEA4) or seed development (O2). FEA4 is expressed in all four tissues while KN1 is only expressed in SAM and seed, and O2 is only expressed in seed. The performance of the GRNs were evaluated by TF ChIP-Seq data using area under the receiver operator characteristic curves (AUROC) and area under the precision-recall curves (AUPR). These are widely used summary statistics for binary classification problems, such that values higher than those obtained using random samples indicate that the classification algorithm has detected more patterns than expected for a random subset. From each ChIP-Seq dataset, genes with high-confidence peaks within 10 kb regions were considered as positive targets for that transcription factor (see Methods for details). The expression patterns for KN1, FEA4 and O2 were consistent with the published gene expression atlas [44] for these three TFs, and the summary statistics for all of our GRNs were all better than random samples except for O2 (Table 2). It has been shown that there is a very low overlap between O2-bound genes and genes that are misregulated in *o2* mutants [43]. Thus, it may be difficult for any algorithm that is purely based on expression data to infer regulatory interactions for O2. Aside from the O2 GRN, the AUROC and AUPR values suggested that our tissue-specific GRNs predicted regulatory interactions that were consistent with ChIP-Seq data. Although GENIE3 generally resulted in similar AUROC and AUPR values compared to MRNET and CLR, the AUROC and AUPR values for FEA4 SAM networks generated by GENIE3 were higher than the MRNET and CLR networks (Additional file 3). Since it’s already known that FEA4 is an important regulator in SAM, we chose the GENIE3 as our network construction method for additional experiments.

**Table 2:** Evaluation of tissue GRNs generated by GENIE3

KN1	AUROC	AUPR	FEA4	AUROC	AUPR	O2	AUROC	AUPR
Random	0.500	0.108	random	0.500	0.103	random	0.500	0.061
SAM	0.558	0.187	leaf	0.545	0.147	seed	0.496	0.07
Seed	0.554	0.193	root	0.541	0.14			
			SAM	0.56	0.171			
			seed	0.533	0.15			

AUROC and AUPR values were calculated tissue GRNs using three TFs’ ChIP-Seq data (KN1, FEA4 and O2). The random networks (random) were permutated 10,000 times. The leaf, root, SAM and seed refer to tissue-specific GRN

In the next set of analyses, for normalization purposes, the four GRNs were constrained to include only the top 1 million interactions (edges) calculated by GENIE3. This is a commonly used cutoff for networks [27] and allows us to compare networks between tissues with different total number of edges. For all remaining results, unless specifically indicated, otherwise the GRNs used for analysis are constrained in this manner. Edges of networks were treated as “directed” wherein TFs were modeled as regulators and all genes expressed in that tissue as targets. We compared the edge overlap among four tissues (Additional file 4). For pairwise comparison, leaf and SAM GRNs shared the most of edges with 7.12% (71,190/100000) common between the two tissues, followed by seed and SAM 5.07% (50,664/1000000). To our surprise, of four million edges total, about 80% of edges were unique to a tissue and only 0.268% (2679 /1000000) edges were shared between all four tissues. This result indicated that even though over 50% of TFs are expressed in four tissues, there are likely distinct regulatory targets in different specific tissues. We investigated the 2679 shared edges of four GRNs consisting of 353 TFs and 1657 target genes (Additional file 4 & Additional file 5). The GO analysis of target genes revealed multiple essential biological processes including cell cycle, DNA replication, cell division and chromosome organization (Additional file 6). Interestingly, there were 30 genes annotated as histone H3K9 methylation (*p*-value = 1.04E-21) which suggested the importance of epigenetic regulation, and particularly heterochromatin formation and gene silencing [45–47]. These interactions may be necessary for plant growth.

### GRN analysis can be used to predict tissue-specific regulation by TFs

**Fig. 2:**
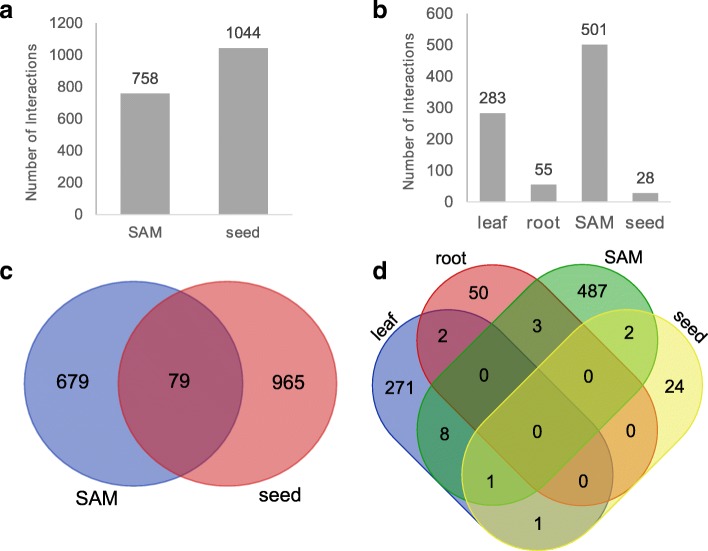
Target prediction of top 1 million edges for Knotted1 (KN1) (**a** and **c**) and Fasciated ear4 (FEA4) (**b** and **d**) in different tissues. **a** Number of predicted KN1 targets in each tissue-specific GRN. **b** Number of predicted FEA4 targets in each tissue-specific GRN. **c** A Venn diagram showing the overlap of KN1 targets between the SAM and seed GRN. **d** A Venn diagram showing the overlap of FEA4 targets between the four tissue-specific GRNs

**Fig. 3:**
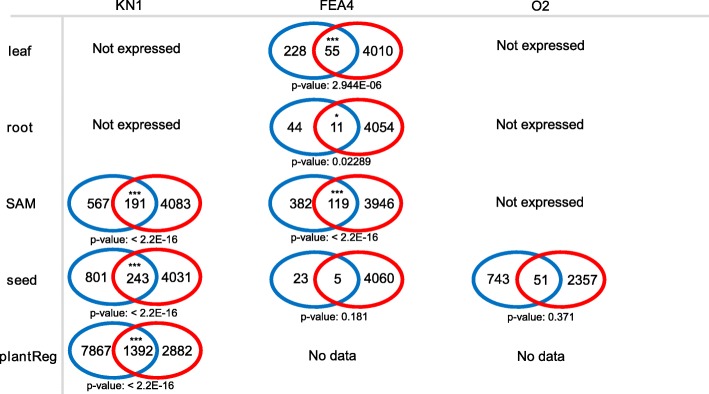
Venn diagrams summarizing the overlap between predicted targets and ChIP-Seq identified targets of KN1, FEA4 and O2. Blue circles are the number of predicted targets for tissue-specific GRN (leaf, root, SAM and seed) or the Plant Transcriptional Regulatory Map (PlantReg); red circles are the number of targets identified by ChIP-Seq. KN1 and O2 were not expressed in some tissues (Not expressed). FEA4 and O2 were not included in PlantReg database (No data). *P*-values were calculated from one-tail Fisher’s exact tests and significant overlaps were indicated with *** (*p*-values less than 0.01) or * (*p*-values less than 0.05)

After exploring the overall network quality, we further analyzed the tissue-specific interactions for KN1, FEA4 and O2. Consistent with their expression pattern, O2 only had predicted interactions in seed, KN1 only had predicted interactions in SAM and seed, while FEA4 had predicted interactions in all four tissues (Figs 2 and 3). For both KN1 and FEA4, SAM GRNs had over 500 predicted interactions. This is consistent with important regulatory roles for KN1 and FEA4 in SAM development, and such functions have been reported for these TFs [41, 42]. For KN1 and FEA4 respectively, 91.23% (1644/1802) and 95.96% (832/867) interactions were predicted to be exclusive for one tissue (Fig. 2c, d). Interestingly, we found two unique GO terms, “shoot system development (p-value: 1.31E-02)” and “nutrient reservoir activity (p-value: 6.31E-25)” from KN1 SAM targets and O2 seed targets respectively (Additional file 7) suggesting that the tissue-specific GRNs identified genes with relevant functionality.

If GRN predictions were enriched for ChIP-Seq confirmed targets, that would suggest that GRNs could reliably identify putative targets for other TFs without ChIP-seq data. From the predicted targets of KN1, FEA4 and O2, we compared how many of them in each tissue were confirmed by ChIP-Seq data (Fig. 3 & Additional file 8). One-tail Fisher’s exact tests were applied to test the significance of the overlap. Most of the predictions exhibited significant enrichment for ChIP-identified targets, except for FEA4 seed and O2 seed predictions (*p*-value > 0.05; Fig. 3). This might be because FEA4 has a limited function in seed. Although O2 seed predicted targets were not enriched in O2-bound genes, our network predicted interactions for 7 of the top 10 most down-regulated genes in *o2* mutants, including 6 zein genes which are well-characterized O2 targets [43].

Another prediction method is to search gene promoter regions for TF-specific *cis*-regulatory element [21, 22, 48, 49]. This method relies on high-quality Position Weight Matrices (PWM) that are only available for limited number of TFs in maize. We compared our predictions with the PlantRegMap database [21] containing KN1, but data was not available for FEA4 and O2.The PlantRegMap’s prediction were also significantly enriched (p-value < 2.2E-16) for KN1 ChIP-binding targets.

**Fig. 4:**
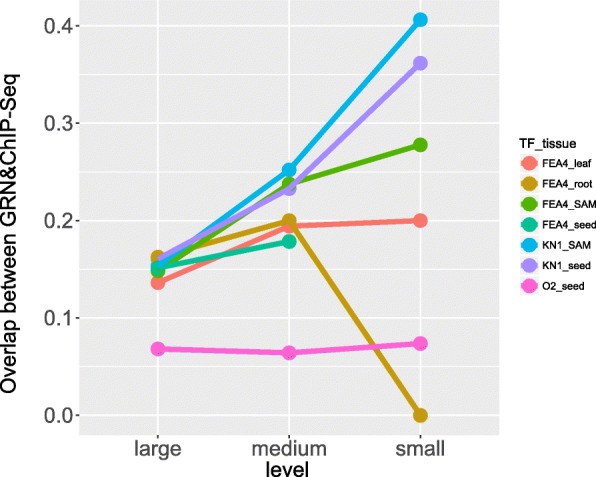
Overlap between ChIP-Seq identified targets and GRN predicted targets in three sizes of networks. Network size was limited to the top 10 million edges (large), top 1 million edges (medium) and top 100,000 edges (small)

Furthermore, we compared the percentage of overlap between GRN predicted targets and ChIP identified targets among various network sizes. Predictions for KN1, FEA4 and O2 that were within 10 million edges were included in the “large” network, only those predictions that were within the top 1 million edges were included in the “medium” networks, and only those predictions in the top 100,000 edges were included in the “small” networks. An increasing pattern of overlap percentage (Fig. 4) was observed for most of TFs as more stringent networks, networks with fewer edges, were selected, except for FEA4 in root. In FEA4 root GRN, no overlap targets could be found (Additional file 8), but this is likely related to the small number of interactions (*n* = 2). Moreover, we compared tissue GRNs with developmental atlas GRNs (Additional file 8) [27]. The atlas GRNs were also created using GENIE3, but used different mRNA and protein expression datasets. 2200 TFs and 545 TFs were included in the mRNA and protein GRN respectively. KN1 and O2 were in both GRNs, but FEA4 only in the mRNA GRN (Additional file 9). We found our tissue-specific GRNs had comparable or better overlap percentage between predicted targets and ChIP-identified targets with the atlas GRNs (Additional file 9). The overlap percentage also increased when using small networks, except FEA4-mRNA GRN. In conclusion, these results demonstrated that tissue-specific GRNs can predict TF binding interactions in different tissues.

### GRN analysis can be used to identify central functionality of TFs in distinct tissues

**Fig. 5:**
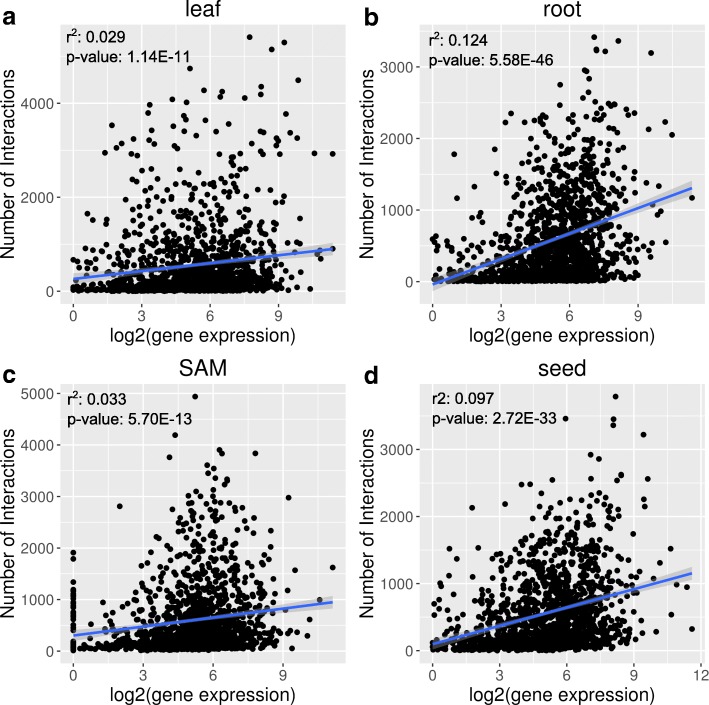
Effect of gene expression (calculated by log2(CPM + 1)) on the number of interactions for TFs in (**a**) Leaf GRN, (**b**) Root GRN, (**c**) SAM GRN, (**d**) Seed GRN. Linear regressions were plotted in blue lines with a grey band as the 95% confidence intervals. R^2^ and p-values were calculated from the linear models by lm() function in R

As we discovered from KN1 and FEA4, TFs may have varied numbers of interactions (degree centrality) in different tissues. We wondered whether this might be correlated with differences in TF gene expression. For example, TFs might have more interactions in the tissue in which the TF is the most highly expressed. To test this, we plotted the number of interactions for each TF against their expression level in each tissue (Fig. 5 & Additional file 10). This analysis included 1406 TFs with at least one interaction in all four tissue GRNs. For all four tissues, the R-squared values were between 0.0012 and 0.124 (Fig. 5 & Additional file 10) for linear regression models of the number of interactions against gene expression (measured by CPM or log2(CPM + 1)). This suggested that there were no linear relationships between TF expression and degree centrality, and that the difference in number of interactions was not likely to be caused by differential TF gene expression. Alternatively, differences in degree centrality may reveal distinct biological function or activity for TFs in different tissues.

**Fig. 6:**
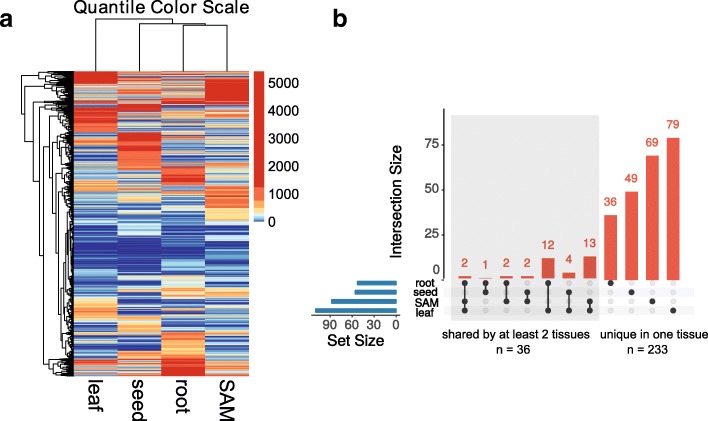
TF interactions in four tissue-specific GRNs. **a** A heatmap showing the distinct pattern on number of targets for each TF among four tissue-specific GRNs. The color scale was based on quantile breaks such that each color represents 10% of the data. Hierarchical clustering was based on Euclidian distances. **b** An UpSet graph visualizing the set interactions among key TFs in each tissue GRN. Number of key TFs expressed in each individual or combination of tissues were represented in bar plot (orange). The intersections were represented by connected black dots. Total number of key TFs expressed in each tissue were represented in blue bar plot. TFs shared by at least two tissues were shaded in light grey

TF degree centrality varied widely for specific TFs between tissues (Fig. 6a). We calculated the coefficient of variation (CV), a measure of relative variability, on degree centrality between the four tissues. The CV ranges from 9.583 to 186.376 with a mean value equales to 88.444. To focus on TFs with large numbers of predicted interactions, a minimum difference in degree centrality of 500 was considered acceptable for further analysis (Additional file 11). We then analyzed the TFs with top 100 largest CVs and found 12 (leaf), 28 (root), 28 (SAM) and 32 (seed) of them had the highest degree centrality in each tissue. TFs with large numbers of interactions in the leaf included genes that would be expected to regulate different aspects of leaf development based on homology to Arabidopsis, like SPL9/15 [50] and TCP2/24 [51]. For example, GRMZM2G126018 (homologous to SPL9/15) has 765 interactions in leaf, but no more than 80 in the other three tissues. We also found GRMZM2G171912 (HY5), GRMZM2G028438 (SCL8) and GRMZM2G146020 (VIP1) had much more interactions in root than other tissues (Additional file 11). Together, these indicated the TFs that appeared important based upon our analyses had other features that were suggestive of unique roles in each tissue.

TFs with a degree centrality > 2000 were defined as key TFs in each tissue, and there were relatively small numbers of these in each tissue (Fig. 6 & Additional file 12). There were 110 key TFs in leaf, 53 in root, 88 in SAM and 56 in seed (Additional file 13). A few well-studied examples included Rough sheath2 (RS2, GRMZM2G403620) in leaf, Homobox3 (HOX3, GRMZM2G314546) in SAM and Prolamin-box binding factor1 (PBF1, GRMZM2G146283) in seed. An overlap visualization (Fig. 6b) showed that 75.90% (233/307) key TFs were unique to one tissue. We also found 36 key TFs that were shared by at least two tissues (Additional file 14). In-depth mining using BioMart (http://plants.ensembl.org) revealed that the homologs of these 36 TFs were critical for development in Arabidopsis (Additional file 14). One example is BZIP113 (GRMZM2G445575) which is homologous to TGACG motif-binding factor (TGA) family in Arabidopsis. It has been shown that TGA genes involved in flowering [52], biotic stress [53] and nitrogen signaling [54]. These 36 TFs are candidates for broad transcriptional regulators in maize. In short, our data suggests that each tissue may use unique TFs as key regulators that can be identified using network analysis.

### Topological and clustering analysis

To characterize the topology of the tissue-specific GRNs, each network’s topological characteristics were computed by NetworkAnalyzer [55]. It has been shown that robust biological networks tend to have scale-free architectures which fit a power-law distribution [55, 56]. Since GRNs are directed networks (TFs regulate target genes), only out-node degree distributions were calculated. For all four tissue GRNs, the connectivity of out-node distributions fit the power-law with R-squared values ranging from 0.398 to 0.601 (Additional file 15), suggesting that our GRNs were robust. These R-squared values are lower than what was determined for our previous optimized maize GCN. This is likely related to the fact that this is a GRN using TFs as regulators, which tend to have higher numbers of interactions than what will be predicted by the power-law distribution.

**Fig. 7:**
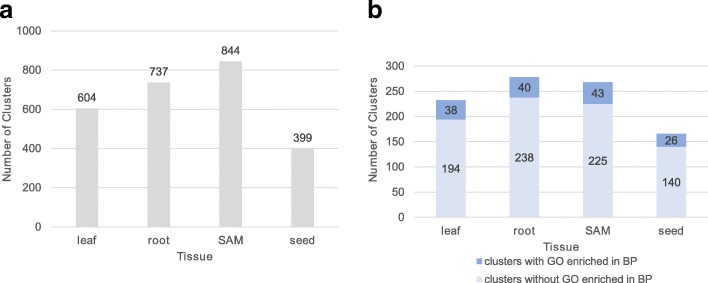
A summary of Markov Cluster Algorithm (MCL) clustering. **a** Total number of clusters discovered by MCL in each tissue-specific GRN. **b** Number of clusters with more than 10 genes. Clusters with at least one significant Gene Ontology (GO) term in Biological Process (BP) were colored dark blue. Clusters without significant Gene Ontology (GO) term in Biological Process (BP) were colored light blue

Next, to find functional modules, GRNs were partitioned using the Markov Cluster Algorithm (MCL) [57], an unsupervised clustering algorithm based on network topology. This method has been successfully applied to maize and other plant species [58–61]. The MCL differentiated 604, 737, 844, 399 modules in leaf, root, SAM and seed, suggesting these were functional modules in these tissues (Fig. 7). Among those modules, 232 (leaf GRN), 278 (root GRN), 268 (SAM GRN) and 166 (seed GRN) of them had more than 10 genes (Fig. 7) and were therefore amenable to GO analysis. We did GO enrichment analysis for these 944 modules using g:profile [62]. We found 156 modules that were enriched for at least one Biological Process (BP) GO term. In each tissue, the largest module was enriched for genes that would be likely to support the biology of that tissue: photosynthesis (leaf), translation (root), protein catabolic process (SAM) and cellular amino acid metabolic process (seed). A deeper look at the largest module in leaf showed enrichment in generation of precursor metabolites and energy (*p*-value = 7.65E-08), carotenoid biosynthetic process (p-value = 1.59E-04) and response to blue light (p-value = 5.87E-04) (Additional file 16). This indicated that MCL could recover biologically relevant modules in each tissue. Gene lists of modules in each tissue and their GO enrichment can be downloaded from our website.

### Website design

**Fig. 8:**
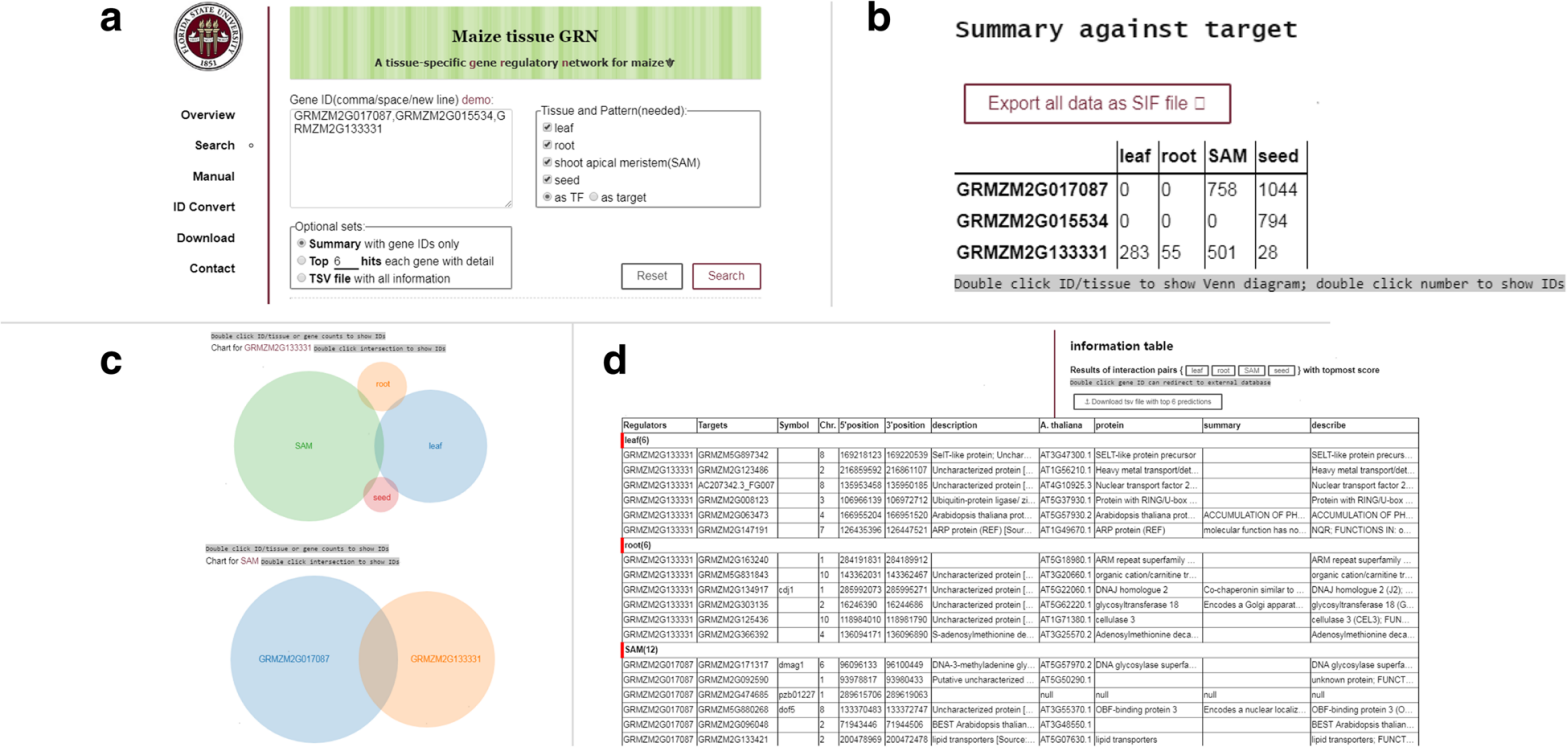
Website screenshots. **a** Search page with all selectable parameters. **b** Summary table queried by KN1(GRM2G017087), FEA4 (GRMZM2G133331) and O2 (GRMZM2G015534). **c** Screenshot of interactive Venn diagram. **d** Screenshot of table with details showing top 6 targets from each tissue queried by KN1, FEA4 and O2

To share our results, we constructed a user-friendly web portal, Maize tissue-specific Gene Regulatory Network (mGRN, http://www.bio.fsu.edu/mcginnislab/mgrn) using MySQL and PHP. It provides search, visualization and download services (Fig. 8). Users can search for TF regulated targets (as TF) or TF regulators (as target) for query genes in four tissues-specific GRNs (Fig. 8a). By default, a summary table of number of predicted regulatory interactions is provided (Fig. 8b). Double clicking the numbers will link to the gene IDs in each category. If there are two to four intersections, double clicking the tissue or genes will launch an interactive Venn diagram (Fig. 8c). Overlap gene IDs and number of genes can be retrieved from the plot. A detailed tutorial is provided on how to visualize our network result in Cytoscape and R (Additional file 17).

The top target genes with detailed information can be fetched on web (Fig. 8d). The TF regulator with predicted targets are the first two columns. To better understand gene functions, targets or regulators gene positions and descriptions based on AGPv3.31, as well as the best matched Arabidopsis gene annotation from BLASTP are provided. Double clicking “Regulator”, “Targets” or “A.thaliana” gene IDs redirects to GRASSIUS [5], MaizeGDB [63] or Araport [64] respectively for easy mining. All search result as well as whole networks can be downloaded from the website for further analysis. So far, our database only accepts maize version 3 gene IDs. An “ID Convert” tool is set up for converting between maize version 4 gene IDs and version 3 gene IDs.

## Discussion

The maize gene expression atlas [34] describes some level of tissue-specific expression for over half of the genes in maize. This suggests that tissue specificity of gene expression is biologically important. In this study, we have constructed maize GRNs from RNA-Seq expression data for leaf, root, SAM and seed tissue using a machine learning algorithm, GENIE3. There are other GRN construction methods, but GENIE3 was selected for analysis because it only requires gene expression data and does not require assumptions on the data distribution. Bayesian-based methods, such as BNFinder [65], need delicate genetic perturbation or time-series design. Both are hard to obtain from public data. GENIE3 also has the ability to reveal non-linear relationships between TF and target where Pearson correlation coefficient (PCC) and Spearman correlation coefficient (SCC) based methods detect linear relationships [66]. Third, unlike Bayesian methods, GENIE3 can discover feedback loops which are prevalent in biological networks. For example, the feedback loop between *CLAVATA* and *WUSCHEL* in controlling SAM development has been reported in both Arabidopsis and maize [67–69]. Unlike correlation and mutual information (MI) based methods GENIE3 predicts the direction of the regulations since it generates two values for modeling gene *i* to *j* and *j* to *i*. The GENIE3 authors showed their methods were significant better than MRNET and CLR when taking directionality into account [24], and GENIE3 was the best performer of the DREAM5 challenge based on the unbiased evaluation from an independent group of evaluators [25]. When we compared GENIE3 with two MI-based methods, MRNET and CLR, there were minor differences in AUROC and AUPR values (Additional file 3) which did not outweigh the advantages to using GENIE3.

Using publicly available RNA-Seq data, we predicted tissue-specific TF interactions at a similar positive rate with an atlas GRN study [27]. Our GRNs performed well based upon evaluation with TF ChIP-Seq data. This study generated GRNs with 2241 TFs and provided a high enough level of resolution to reveal the spatial variation of gene regulation.

In our analysis, we found 80% of interactions are unique to one tissue in maize although over 80% of genes and over 50% of TFs are expressed in all four tissues. Furthermore, TF expression did not correlate with the number of interactions. This indicates that interactions between genes may provide a mechanism for tissue-specific functions that cannot be explained with variability in gene expression alone. This correlates well with a recent study in human suggesting that TFs have uniform expression but differential binding targets to support tissue-specific functions [70]. It has been previously reported that transcriptional regulatory networks in humans can be cell-type specific [71].

Compared with previous large-scale studies in maize, we utilized networks to elucidate gene regulation among multiple tissues and confirmed known functional patterns that had been previously published for some transcription factors [3, 72]. For example, the TF Myb-related protein-1 (MRP-1) was only included in our seed-specific GRN, and our network included 51 of the 93 genes predicted to be regulated by MRP-1 in another study [4].This confirms that network analysis can be used to discover tissue-specific TF interactions. We also compared our network predictions with PlantRegMap and found they were both enriched for ChIP-confirmed targets. By inferring GRNs from expression data, we do not rely on high-quality PWMs as PlantRegMap does, and so predictions can be extended to a larger number of TFs. Moreover, by using expression data from multiple libraries, we can focus on spatial and/or temporal specific regulatory interactions.

## Conclusions

In this study, we constructed four tissue-specific GRNs for maize, including leaf, root, SAM and seed. The quality of these GRNs was assessed using comparisons with experimental data and biological functional enrichment. These assessments suggest that the tissue-specific GRNs predict high-confidence TF regulatory targets. We provided examples of TF-target interactions predicted to have tissue-specific function. Functional modules were also identified and can provide gene cluster information at the tissue level. To our knowledge, this is the first systematic study in maize on TF regulatory networks in different tissues, and it demonstrates that using statistical methods to infer GRNs can expand our knowledge of gene regulation and circumvent the limitations of some genomic techniques in plants. To make our findings more accessible, a mGRN web database (http://www.bio.fsu.edu/mcginnislab/mgrn) was built that includes gene functions and links to other web portals. We hope our results can facilitate further gene regulatory research. Moreover, our framework to construct tissue-specific GRNs could also be applied to other organisms with abundant genome-wide expression data.

## Methods

### RNA-Seq data collection and process

The RNA-Seq libraries were processed as described previously [33]. In brief, RNA-Seq samples were downloaded from NCBI SRA [73] and converted to fastq format by fastq-dump command in SRA Toolkit 2.5.2. Adapters were trimmed by Cutadapt 1.8.1 [74]. Then reads were aligned to maize genome AGPv3 (Ensembl Plant release 31) by HISAT2 v2.0.4 [75]. Gene-level expression were measured by FeatureCounts 1.5.0 [76], then normalized by Trimmed Mean of M-values (TMM) [77] and reported in log2 normalized Counts Per Million (CPM). In summary, 394 (leaf), 176 (root), 406 (SAM) and 159 (seed) libraries were analyzed for each tissue.

### Network construction

To filter lowly expressed genes, genes with less than 1 CPM in more than 10% of libraries in each tissue were excluded. The GEne Network Inference with Ensemble of trees (GENIE3) algorithm [24] was used to construct tissue-specific Gene Regulatory Networks (GRNs), more specifically, the version that implemented in R and C. 2587 TFs in maize genome from GRASSIUS [5] were specified as candidate regulators.

### ChIP-Seq identified targets and network evaluation

For KN1 and FEA4, the ChIP-Seq targets were downloaded from their original papers [41, 42]. For O2, the ChIP-Seq summit file was downloaded from Gene Expression Omnibus (http://www.ncbi.nlm.nih.gov/geo) under accession number GSE39161. To keep the criteria the same as KN1 and FEA4, genes within 10 kb of peak summits were defined as ChIP-Seq identified targets.

We used Area Under an ROC Curve (AUROC) and Area Under a Precision-Recall Curve (AUPR) to evaluate network quality. KN1, FEA4 and O2 ChIP-Seq identified targets were used as positive set. Values were calculated by PRROC package in R [78]. To generate random networks, target genes were permuted 10,000 times and the correspondent AUROC and AUPR values were averaged. One-tail Fisher’s exact tests were calculated using fisher.test() function in R. The atlas mRNA and protein GRNs were downloaded from the original paper [27]. The list of KN1 targets predicted by PlantRegMap was downloaded from its website (http://plantregmap.cbi.pku.edu.cn). Venn diagrams were plotted by Venn (http://bioinformatics.psb.ugent.be/webtools/Venn/). UpSet graphs were plotted by Intervene [79].

### Gene ontology enrichment and homology analysis

GO enrichment was analyzed by Gene Group Functional Profiling (GOST) tool from g:Profiler (version Ensembl Genomes 31) [62]. *P*-values were calculated from Fisher’s one-tail test and corrected by Set Counts and Sizes (SCS) method for multiple testing. Only GO terms with P-values less than 0.05 were reported. Arabidopsis homologs were retrieved from BioMart (Ensembl Genomes 31).

### Module detection and characterization

Network topological characterization for tissue GRNs (top 1 million edges) were computed by NetworkAnalyzer [55] in Cytocscape [80]. Modules were detected by Markov Cluster Algorithm (MCL) [57] with inflation values set to 2.5. List of genes were read into R and analyzed by gProfileR (https://cran.r-project.org/web/packages/gProfileR) package for GO enrichment.

### Website design

The web database (https://www.bio.fsu.edu/mcginnislab/mgrn/) is built using MySQL and PHP. The maize gene description was retrieved from BioMart on Ensembl Plant release 31. Gene symbol is based on annotation on MaizeGDB. The Arabidopsis gene description was downloaded from TAIR10. BLASTP was done using local BLAST+ 2.2.28 [81]. The maize gene ID conversion was downloaded from Gramene (ftp://ftp.gramene.org/pub/gramene/archives/PAST_RELEASES/release-56/gff3/zea_mays/gene_id_mapping_v3_to_v4/). All data and source code is available at Github (https://github.com/timedreamer/maize_tissue-specific_GRN).

## Additional files

Additional file 1: RNA-Seq libraries used in this analysis. (XLSX 71 kb)

Additional file 2: GO enrichment analysis for tissue-specific genes in four tissue GRNs. (XLSX 23 kb)

Additional file 3: Evaluation of tissue GRNs generated by MRNET and CLR. (XLSX 9 kb)

Additional file 4: A Venn diagram showing the overlap among top 1 million edges of each tissue-specific GRN. (PDF 55 kb)

Additional file 5: The 353 TFs and 1657 targets included in the 2679 edges shared by four tissue GRNs. (XLSX 70 kb)

Additional file 6: GO enrichment analysis for 1657 conserved targets in four tissue GRNs. (XLSX 22 kb)

Additional file 7: GO enrichment analysis for KN1, FEA4 and O2 targets in four tissue GRNs. (XLSX 19 kb)

Additional file 8: Predicted TF target overlap with ChIP-Seq confirmed binding genes for KN1, FEA4 and O2. The leaf, root, SAM and seed refer to our tissue GRNs. “Protein” and “RNA” refer to protein GRN and RNA GRN from Walley et al. (2016) dataset. “Large network” used top 10 million edges in KN1, FEA4 and O2 networks. “Medium network” used top 1 million edges, while “Small network” used top 100,000 edges. “Atlas GRN medium” used top 1 million edges from Walley et al. (2016) dataset while “Atlas GRN small” used top 100,000 edges. (XLSX 13 kb)

Additional file 9: Comparison of tissue-specific GRNs and atlas GRNs on percentage of overlap between GRN predicted targets and ChIP-Seq identified targets. Leaf, root, SAM and seed GRNs are networks in this study. mRNA and protein networks were constructed by Walley et al. Medium networks (light grey) are the targets within top 1 million edges. Small networks (dark grey) are the targets within top 100,000 edges. (PDF 77 kb)

Additional file 10: Effect of gene expression (calculated by CPM) on the number of interactions for TFs in (A) Leaf GRN, (B) Root GRN, (C) SAM GRN, (D) Seed GRN. Linear regressions were plotted in blue lines with a grey band as the 95% confidence intervals. R^2^ and *p*-values were calculated from the linear models by lm() function in R. (PDF 914 kb)

Additional file 11: Predicted TF targets from top 1 million edges in each tissue. “max _tissue” means which tissue has the highest number of interactions. “CV” is the coefficient of variance. (XLSX 76 kb)

Additional file 12: Degree centrality (Number of targets) of top 1 million edges for TFs in (A) Leaf GRN, (B) Root GRN, (C) SAM GRN and (D) Seed GRM. Red lines showing TF with degree centrality > 2000. (PDF 338 kb)

Additional file 13: Key TFs in four tissues. (XLSX 12 kb)

Additional file 14: Homologs of shared TF by more than two tissues in Arabidopsis. Genes without homologs identified by BioMart were left blank. (XLSX 11 kb)

Additional file 15: Average neighborhood connectivity for four tissue GRNs. The average neighborhood connectivity distribution of all TFs was plotted against number of neighbors. In each network, the top 1 million edges were selected. Red curves show the power-law fitted distribution. R^2^ values indicate the fitness with the power-law model. (PDF 2647 kb)

Additional file 16: GO enrichment analysis for the largest module in leaf detected by MCL. (XLSX 17 kb)

Additional file 17: A short tutorial on visualize mGRN data in Cytoscape and R. (HTML 3228 kb)

## Abbreviations

AUPR: Area under the precision-recall curves
AUROC: Area under the receiver operator characteristic curves
ChIP-Seq: Chromatin immunoprecipitation with sequencing
CLR: Context likelihood of relatedness
CPM: Counts per million
CV: Coefficient of variation
DAP-seq: DNA affinity purification sequencing
GENIE3: Gene network inference with ensemble of trees
GO: Gene ontology
GRASSIUS: Grass regulatory information services
GRN: Gene regulatory networks
MCL: Markov cluster algorithm
mGRN: Maize tissue-specific gene regulatory network
MI: Mutual information
MRNET: Minimum redundancy network
PBM: Protein binding microarrays
PCC: Pearson correlation coefficient
PWM: Position weighed matrix
SAM: Shoot apical meristem
SCC: Spearman correlation coefficient
SELEX: Systematic evolution of ligands by exponential enrichment
