# Supplementary figures and images for: Distinct tissue-specific transcriptional regulation revealed by gene regulatory networks in maize
Source: BMC Plant Biol. 2018 Jun 7;18:111. doi: 10.1186/s12870-018-1329-y (PMC6040155; doi:10.1186/s12870-018-1329-y)

Additional file 4

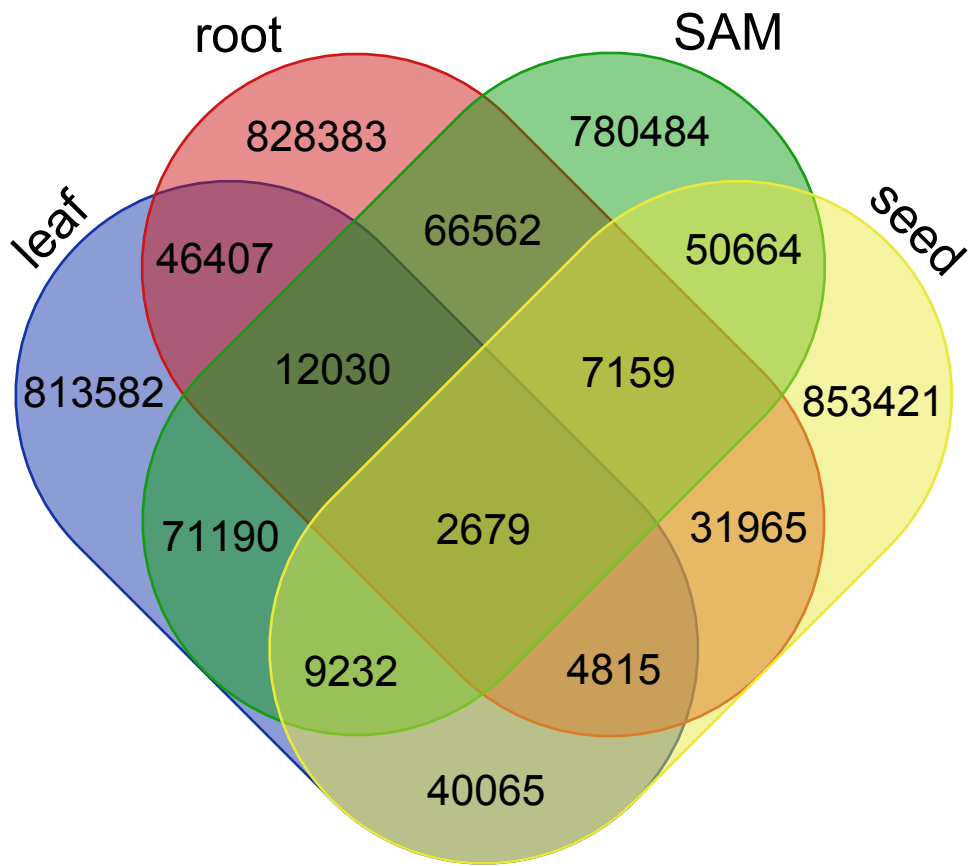

Supplement: Supplementary file 4 — A Venn diagram showing the overlap among top 1 million edges of each tissue-specific GRN. (PDF 55 kb) [file 12870_2018_1329_MOESM4_ESM.pdf]

Additional file 9

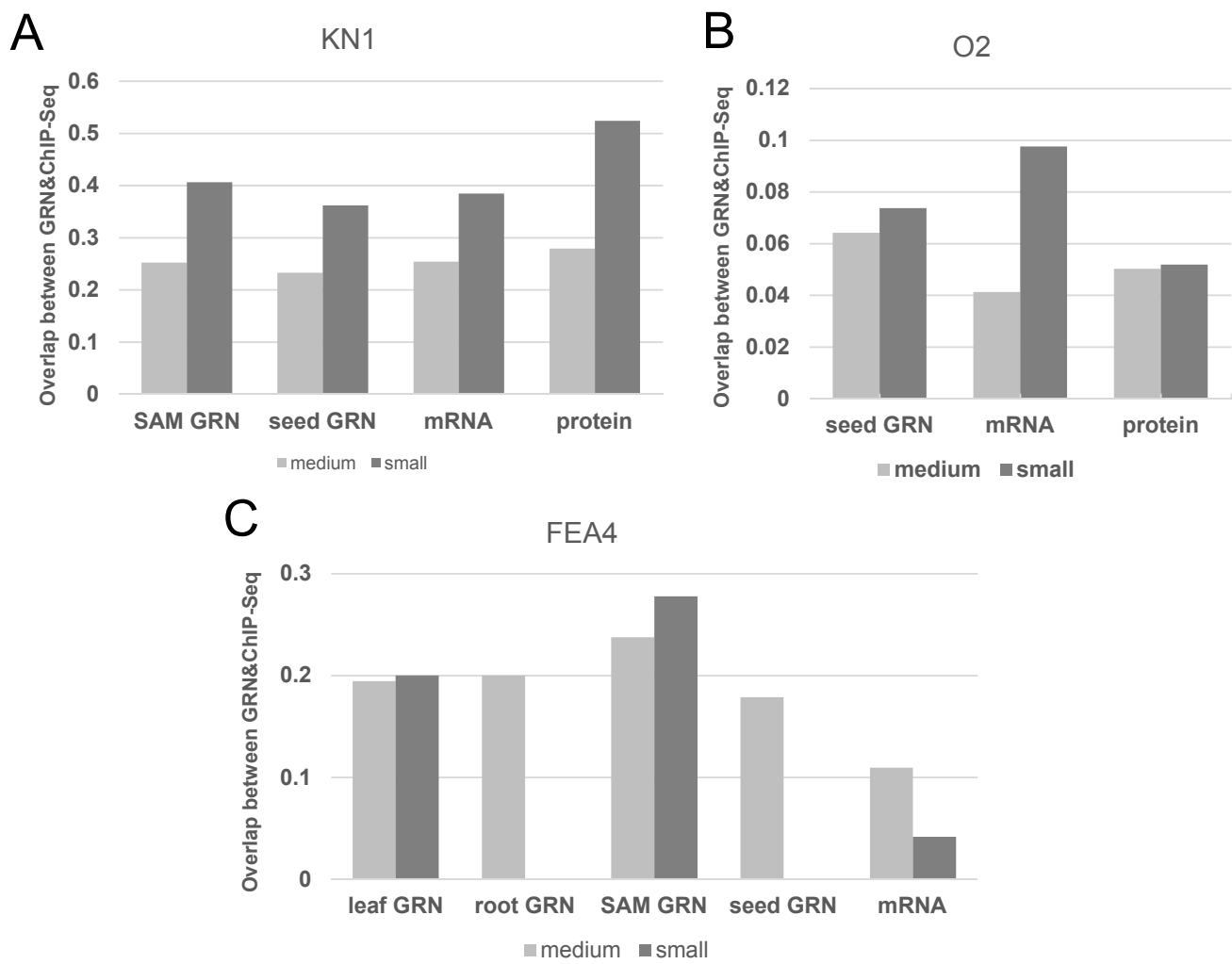

Supplement: Supplementary file 9 — Comparison of tissue-specific GRNs and atlas GRNs on percentage of overlap between GRN predicted targets and ChIP-Seq identified targets. Leaf, root, SAM and seed GRNs are networks in this study. mRNA and protein networks were constructed by Walley et al. Medium networks (light grey) are the targets within top 1 million edges. Small networks (dark grey) are the targets within top 100,000 edges. (PDF 77 kb) [file 12870_2018_1329_MOESM9_ESM.pdf]

Additional file 10

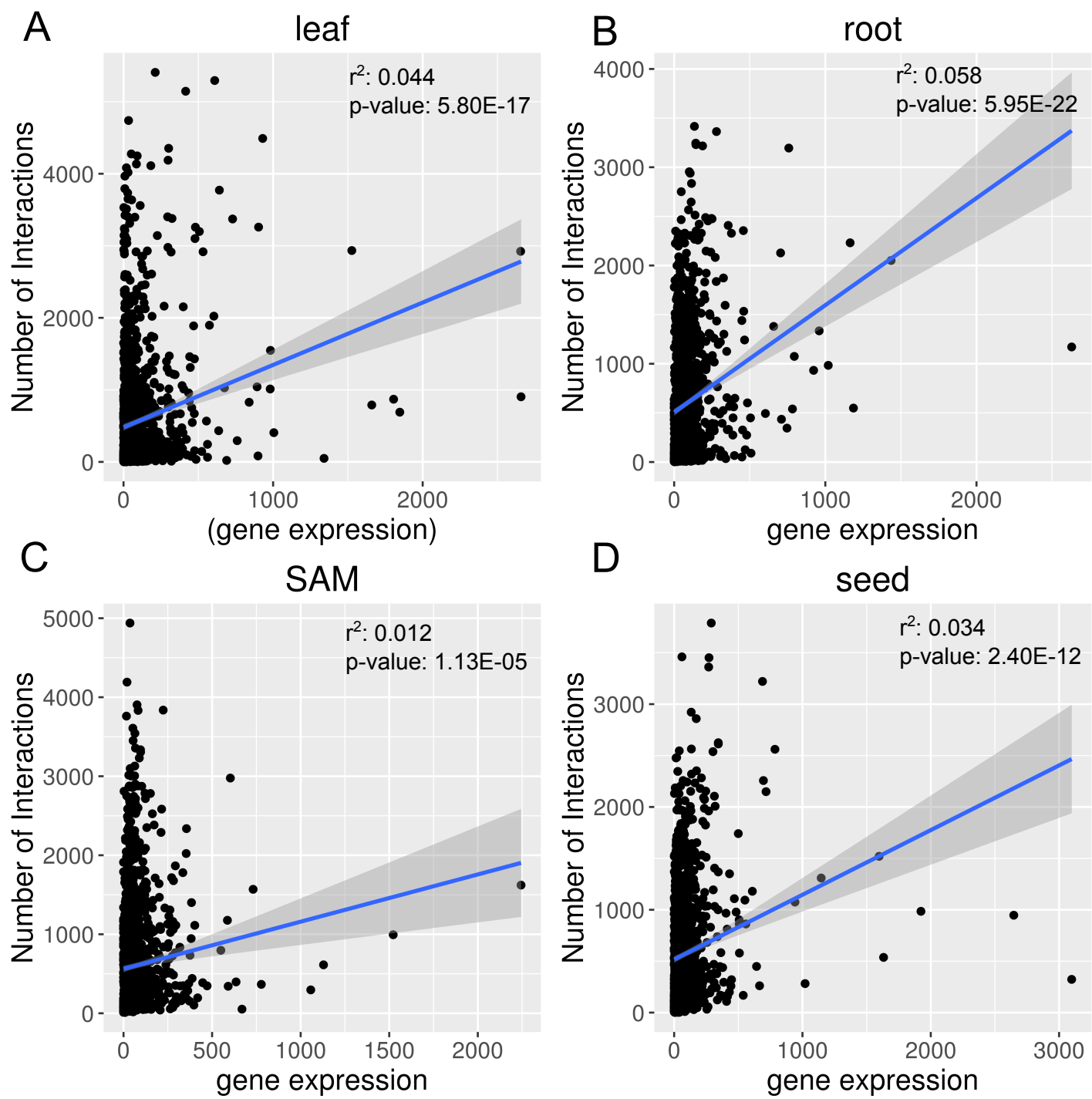

Supplement: Supplementary file 10 — Effect of gene expression (calculated by CPM) on the number of interactions for TFs in (A) Leaf GRN, (B) Root GRN, (C) SAM GRN, (D) Seed GRN. Linear regressions were plotted in blue lines with a grey band as the 95% confidence intervals. R2 and p-values were calculated from the linear models by lm() function in R. (PDF 914 kb) [file 12870_2018_1329_MOESM10_ESM.pdf]

# Additional file 12

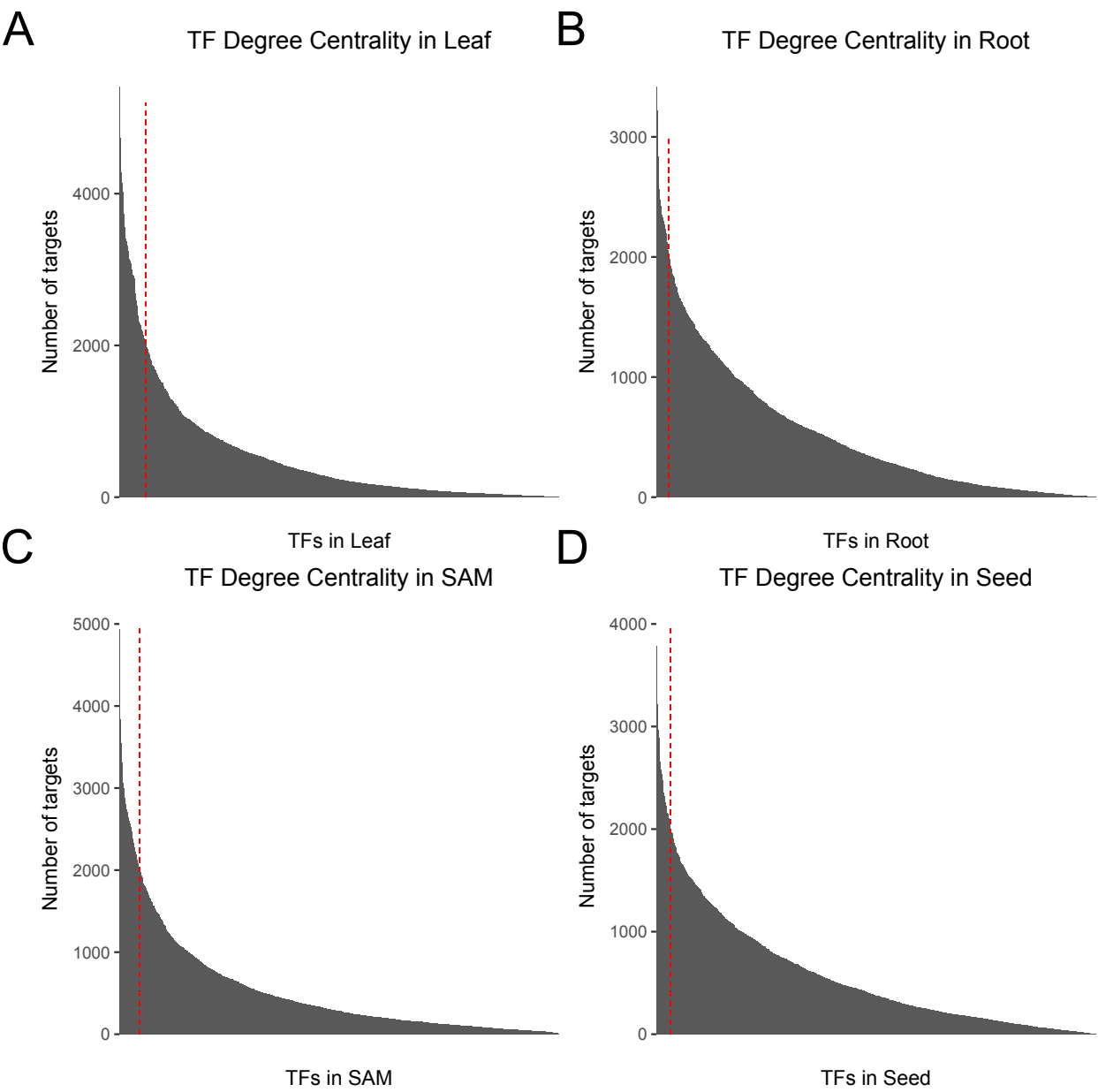

Supplement: Supplementary file 12 — Degree centrality (Number of targets) of top 1 million edges for TFs in (A) Leaf GRN, (B) Root GRN, (C) SAM GRN and (D) Seed GRM. Red lines showing TF with degree centrality > 2000. (PDF 338 kb) [file 12870_2018_1329_MOESM12_ESM.pdf]

# Additional file 15

A

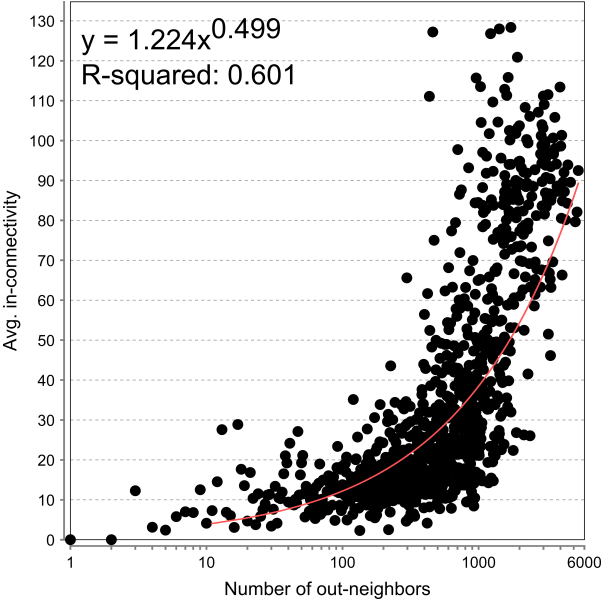

B

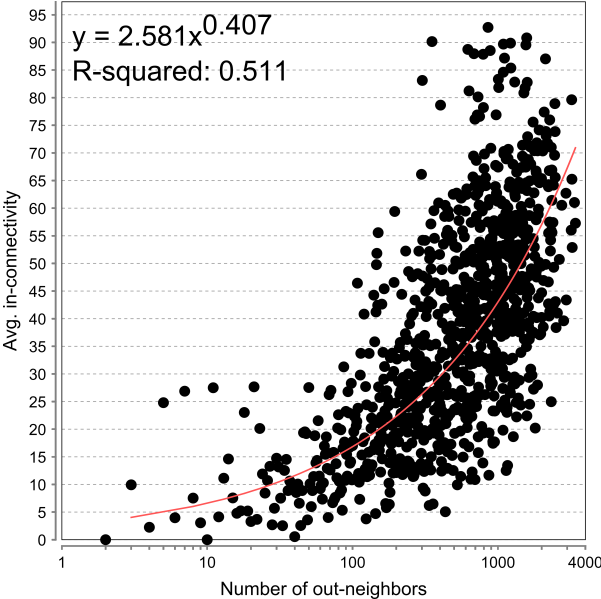

C

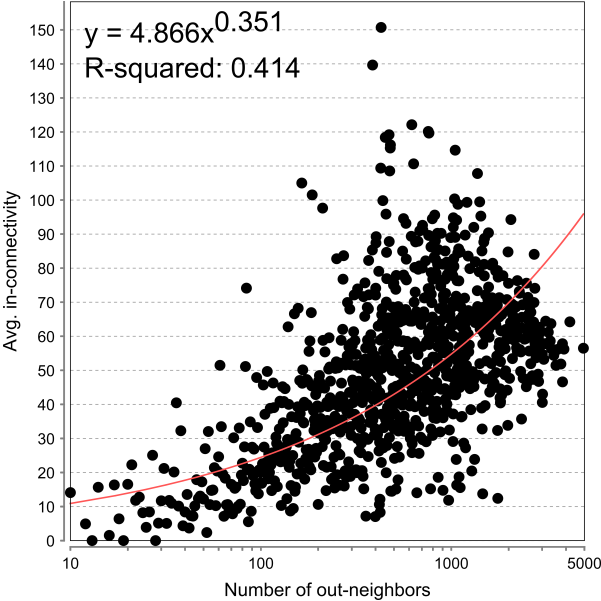

D

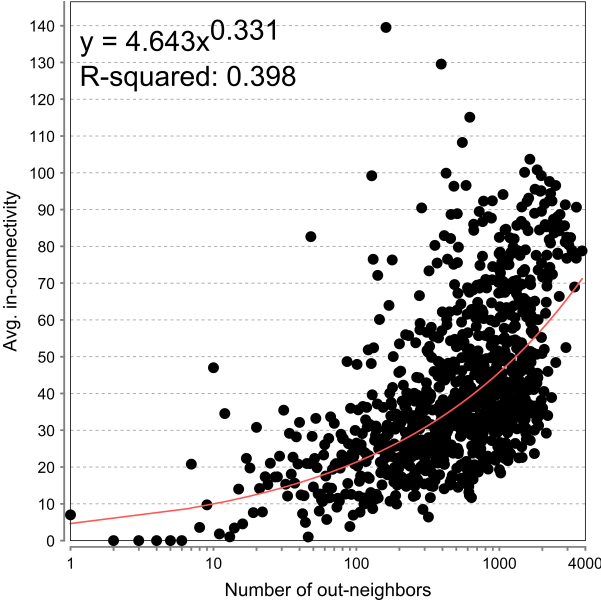

Supplement: Supplementary file 15 — Average neighborhood connectivity for four tissue GRNs. The average neighborhood connectivity distribution of all TFs was plotted against number of neighbors. In each network, the top 1 million edges were selected. Red curves show the power-law fitted distribution. R2 values indicate the fitness with the power-law model. (PDF 2647 kb) [file 12870_2018_1329_MOESM15_ESM.pdf]
