# Supplementary material for: Distinct tissue-specific transcriptional regulation revealed by gene regulatory networks in maize
Source: BMC Plant Biol. 2018 Jun 7;18:111. doi: 10.1186/s12870-018-1329-y (PMC6040155; doi:10.1186/s12870-018-1329-y)
Supplement: Supplementary file 17 — A short tutorial on visualize mGRN data in Cytoscape and R. (HTML 3228 kb) [file 12870_2018_1329_MOESM17_ESM.html]

GRN visualization


# GRN visualization

#### *Ji Huang*

#### *4/10/2018*

## Part 1: Network Visualization in Cytoscape

This part of the tutorial shows how to visualize our GRN result in Cytoscape. Cytoscape is a popular platform for network analysis and visualiztion. It’s freely available on Linux, Mac and Windows.

### 1.1 Download and install Cytoscape.

Please download and install the version for your operating system from the website. We tested on version 3.4.0.

### 1.2 Download the network file.

Cytoscape has the capacity to show text-rich informations such as “Gene description”. Thus, we recommend users download the **TSV file with all information** (Figure 1). Multiple TFs/tissues can be downloaded, but may need extra text processing.

Figure1

The **TSV** file can be opened by any text editors or Excel. See the Excel screenshot (Figure 2). The extra second line **SAM:** can be deleted. Here we kept the line and will show you how to omit that extra line in Cytoscape.

Figure2

### 1.3 Import the network into Cytoscape.

Next, open the Cytoscape and click **Import Network From File** (Figure 3). Select the downloaded network file.

Figure3

In the pop up window (Figure 4), select first column as *Source Node*, second column as *Target Node*, and the rest of columns as *Target Node Attribute*.

Figure4

If you didn’t delete the extra line in **1.2**, you can click **Advance Options** and set the *Ignore lines starting with:* to **SAM** (Figure 5).

Figure5

After this, you should be able to view you network on default settings (Figure 6), although not perfect.

Figure6

### 1.4 Adjust network views.

Most of adjustment for visualization are in the *Control Panel*. We like the **Solid** style (Figure 7).

Figure7

Now the network looks much better. You can also see the detailed node information in the *Table* below (Figure 8).

Figure8

In addition, you can also view multiple TFs’ networks at the same tissue by downloading **Summary with gene IDs only** and then importing into Cytoscape. Here is an example showing the KN1 and FEA4 SAM networks (Figure 9).

Figure9

## Part 2: Visualize networks in R

This part of the tutorial is to show how to visualize our GRN results in R.

### 2.1 Import data and libraries.

To visualize networks in R, we only need the SIF file which can be downloaded by choosing **Summary with gene IDs only** –> **Export all data as SIF file** (Figure 10).

Figure10

```
library(tidyverse)
library(igraph)

# load the downloaded tsv file.

grn <- read_tsv("../data/grn_table.sif", skip = 1,
                col_names = c("regulator", "tissue", "target"))
```

### 2.2 Prepare igraph format for each FEA4 tissue-specific network

We use **FEA** (GRMZM2G133331) as an example. It has predicted targets in all four tissues.

```
FEA4 <- "GRMZM2G133331"

grn_fea4_leaf <- grn %>% 
  filter(regulator == FEA4, tissue == "leaf") %>% 
  select(-tissue) %>% 
  graph_from_data_frame(., directed = T)

grn_fea4_root <- grn %>% 
  filter(regulator == FEA4, tissue == "root") %>% 
  select(-tissue) %>% 
  graph_from_data_frame(., directed = T)

grn_fea4_sam <- grn %>% 
  filter(regulator == FEA4, tissue == "SAM") %>% 
  select(-tissue) %>% 
  graph_from_data_frame(., directed = T)

grn_fea4_seed <- grn %>% 
  filter(regulator == FEA4, tissue == "seed") %>% 
  select(-tissue) %>% 
  graph_from_data_frame(., directed = T)
```

### 2.3 Set the FEA4 to red color

```
V(grn_fea4_leaf)[FEA4]$color <- "red"
V(grn_fea4_root)[FEA4]$color <- "red"
V(grn_fea4_sam)[FEA4]$color <- "red"
V(grn_fea4_seed)[FEA4]$color <- "red"
```

### 2.4 Plot four tissue-specific networks for FEA4

```
par(mfrow = c(2,2), mar = c(1,1,1,1), oma = c(2,4,4,4))

plot(grn_fea4_leaf, edge.arrow.size = .4,vertex.label = NA, vertex.size = 5, 
     layout = layout_with_fr, main = "FEA4 leaf")
plot(grn_fea4_root, edge.arrow.size = .4,vertex.label = NA, vertex.size = 5, 
     layout = layout_with_fr, main = "FEA4 root")
plot(grn_fea4_sam, edge.arrow.size = .4,vertex.label = NA, vertex.size = 5, 
     layout = layout_with_fr, main = "FEA4 SAM")
plot(grn_fea4_seed, edge.arrow.size = .4,vertex.label = NA, vertex.size = 5, 
     layout = layout_with_fr, main = "FEA4 seed")
```
